# Integrated bioinformatics analysis reveals that EZH2-rich domains promote transcriptional repression in cervical cancer

**DOI:** 10.17179/excli2022-5029

**Published:** 2022-06-23

**Authors:** Eric G. Salmerón-Bárcenas, Ana Elvira Zacapala-Gómez, Julio Ortiz-Ortiz, Francisco I. Torres-Rojas, Pedro A. Ávila-López

**Affiliations:** 1 Departamento de Biomedicina Molecular, Centro de Investigación y de Estudios Avanzados del Instituto Politécnico Nacional, Ciudad de México 07360, México; 2 Laboratorio de Biomedicina Molecular, Facultad de Ciencias Químico Biológicas, Universidad Autónoma de Guerrero, Chilpancingo 39070, Guerrero, México; 3 Department of Biochemistry and Molecular Genetics, Feinberg School of Medicine, Northwestern University, Chicago, Illinois 60611, USA

**Keywords:** cervical cancer, EZH2-rich domains, silencers, chromatin domains, senescence

## Abstract

Cervical cancer is the third female cancer most common worldwide. The carcinogenic process involves an alteration of the mechanisms associated with transcription. Several studies have reported an oncogenic role of the polycomb complex subunit, EZH2. However, the role of EZH2 in cervical cancer is unknown. Hence, the objective of this study was to determine the role of EZH2 in transcriptional regulation in cervical cancer. The EZH2 expression and the methylation status of its promoter were analyzed in The Cancer Genome Atlas. The EZH2 enrichment profile was analyzed using chromatin immunoprecipitation with massively parallel DNA sequencing data provided by ENCODE project. The chromatin compartments were identified in the 4D Nucleome Data Portal. The functional annotation was examined in Enrichr. We report that EZH2 expression is increased in cervical cancer which is associated with hypomethylation of its promoter. EZH2 is enriched at promoter and distal intergenic regions. We identified that EZH2 defines chromatin domains enriched with H3K27me3 within repressive compartments in the HeLa-S3 cell line. Additionally, high EZH2 expression is associated with the repression of the senescent phenotype in cervical cancer patients. Our results suggest the participation of EZH2 in the generation of domains with a silencer function in cervical cancer, which regulate the expression of genes associated with cellular senescence.

## List of Abbreviations

CC Cervical cancer

ChIP-seq Chromatin immunoprecipitation with massively parallel DNA sequencing

PcG domains Chromatin repressive domains 

EZH2 Enhancer of Zeste Homolog 2 

GEO Gene Expression Omnibus

GEPIA Gene Expression Profiling Interactive Analysis

GSEA Gene Set Enrichment Analysis 

OS Overall survival

PRC2 Polycomb Repressive Complex 2 

RFS Relapse-free survival

RNA-seq RNA sequencing

TCGA The Cancer Genome Atlas

GTEx The Genotype-Tissue Expression

HPA The Human Protein Atlas database

H3K27me3 Trimethylation of lysine 27 in histone H3 

## Introduction

Cervical cancer (CC) is the third female cancer most common with an incidence and mortality of 6.5 % and 7.7 %, respectively (Ferlay et al., 2021[19]). In the United States, 14,480 new cases and 4,290 deaths were estimated in 2021 (Siegel et al., 2021[53]). The progression of CC involves the aberrant genes' expression through transcriptional and epigenetic alterations, such as DNA aberrant methylation, global histone modifications, and histone variants (Dueñas-González et al., 2005[16]; Salmerón-Bárcenas et al., 2021[48]).

Transcriptional regulation is a key process for cell development and cell fate determination. Hence, alterations in transcription promote malignant transformation (Rhee et al., 2017[46]). In this sense, the Polycomb Repressive Complex 2 (PRC2) is a protein complex that mediates gene repression by modulating the chromatin structure (Pasini and Di Croce, 2016[41]). Enhancer of Zeste Homolog 2 (EZH2) is the catalytic subunit of PRC2 with a histone methyltransferase activity that catalyzes the trimethylation of lysine 27 in histone H3 (H3K27me3) (Simon and Lange, 2008[54]). H3K27me3 promotes gene repression and has important functions in cell fate determination and development (Simon and Lange, 2008[54]). It has been reported that EZH2 is an important regulator of the cell cycle, autophagy, apoptosis, cellular senescence, and cancer (Duan et al., 2020[15]). EZH2 is overexpressed in several types of cancer such as breast cancer, esophageal cancer, endometrial carcinoma, gastric cancer, nasopharyngeal carcinoma, prostate cancer, and thyroid carcinoma (Bachmann et al., 2006[2]; Fan et al., 2020[18]; Gan et al., 2018[20]; Krill et al., 2020[26]; Pellecchia et al., 2020[42]; Qiu et al., 2020[44]; Varambally et al., 2002[58]). For instance, EZH2 overexpression in human mammary epithelial cells allows cell invasion and breast cancer aggressiveness (Kleer et al., 2003[25]). In pancreatic cancer, EZH2 overexpression is associated with advanced stages (stage III/IV), and EZH2 knockdown promotes the reduction of proliferation, migration, and invasion (Ma et al., 2018[35]).

Polycomb group proteins are capable of silencing gene expression through chromatin repressive domains (PcG domains), which have H3K27me3 enrichment (deposited by EZH2), and are associated with silencing sequences in the genome called silencers (Schuettengruber et al., 2013[50]). Yichao Cai and colleagues (2021[4]), identified H3K27me3 domains which contact target genes to regulate transcriptional repression (Cai et al., 2021[4]). CRISPR excision of these silencers induces changes in the cellular phenotype such as adhesion, proliferation, and differentiation. Interestingly, the EZH2 inhibition destabilizes the chromatin interactions associated with the silencers, allowing the derepression of cancer-related genes (Cai et al., 2021[4]). Thus, EZH2 is relevant to regulating silencer domains and gene repression. For this reason, it would be important to investigate whether EZH2 is regulating gene repression of cancer-associated genes through silencers.

Here, we identified EZH2 overexpression in CC which is associated with potential diagnostic. Furthermore, this EZH2 increase is associated with hypomethylation of its promoter in CC patients. EZH2 is enriched at promoter and intergenic distal regions in the HeLa-S3 cell line. Interestingly, EZH2 defines chromatin domains (EZH2-rich domains) enriched with H3K27me3, H2A.Z, and CTCF. EZH2-rich domains are enriched in B compartments and are associated with gene silencing, indicating a function associated with silencers. Finally, high EZH2 levels are related with a genetic signature involved with the repression of the senescent phenotype in CC patients. Together, our results suggest the participation of EZH2 in the generation of domains with a silencer function in CC, which regulate the expression of genes associated with cellular senescence.

## Material and Methods

### Expression analysis

The EZH2 expression was analyzed in CC and normal tissue samples from The Cancer Genome Atlas (TCGA) and The Genotype-Tissue Expression (GTEx) datasets using Gene Expression Profiling Interactive Analysis (GEPIA) database (Tang et al., 2017[56]). EZH2 expression was log_2_ (TPM+1) transformed, the differences were calculated using the one-way ANOVA test, and a *p*-*value* ˂0.05 was considered significant. Moreover, EZH2 expression was analyzed in GSE67522 (Sharma et al., 2015[52]) (Platform: GPL10558, Illumina Human HT-12 V4.0 expression BeadChip) and GSE9750 (Scotto et al., 2008[51]) (Platform: GPL96, [HG-U133A] Affymetrix Human Genome U133A Array) datasets from Gene Expression Omnibus (GEO) database (Edgar et al., 2002[17]). The data were analyzed in GEO2R software (Barrett et al., 2013[3]). GSE67522 dataset contains 20 CC and 22 normal tissue samples, and the GSE9750 dataset contains 33 CC and 21 normal tissue samples. The results are shown as median±SD in SigmaPlot version 10.0 (Systat Software, San Jose, CA). The differences were calculated using the *t*-student test and a *p-value *˂0.05 was considered statistically significant. The EZH2 expression to protein level was analyzed by immunohistochemistry assays in CC and cervix/uterine normal tissue samples from the Human Protein Atlas database (HPA) (Uhlén et al., 2015[57]). The EZH2 expression was analyzed in glandular and squamous epithelial cells using anti-EZH2 (CAB009589). Two representative images of CC and normal tissues were randomly selected.

### Methylation analysis

The EZH2 promoter (-2 kb to +2 kb relative to TSS) was downloaded from the Eukaryotic Promoter Database (Périer et al., 1998[43]). The CpG island prediction was performed in the MethPrimer program (Li and Dahiya, 2002[30]). Criteria for predicting CpG islands were a window˃200, shift: 1, Obs/Exp˃0.6, and GC %˃50. The methylation level was analyzed in DiseaseMeth version 2.0 (Xiong et al., 2017[61]). The selected parameters were Disease: Cervical squamous cell carcinoma and endocervical adenocarcinoma (CESC), Gene symbol: EZH2 (NM_152998), Array-based technology: 450k (Illumina Infinium Human Methylation 450 BeadChip), Control type: control from the same tissue/cell lines, Method of differential analysis: *t*-test, Significant *p*-*value*: 0.01, Select region of interest: CGI-related categories, Selected CpG between TSS and TES: CpG island, genomic region: chr7:148580495-148582061.

### Correlation analysis

The correlation analysis between methylation and expression of EZH2 was performed in CC tissue samples from the TCGA dataset using the cBioportal database (Cerami et al., 2012[6]). The correlation was calculated using Spearman, Pearson, and R^2^ coefficients, and a *p*-*value *˂0.05 was considered statistically significant.

### ChIP-seq analysis

To determine the EZH2 enrichment in the HeLa-S3 cell line, we analyzed chromatin immunoprecipitation with massively parallel DNA sequencing (ChIP-seq) data available in ENCODE (Davis et al., 2017[12]). We downloaded raw data corresponding to EZH2 ChIP-seq (ENCFF000BBE and ENCFF000BBD) (Lou et al., 2020[32]; Zhang et al., 2020[65]) and control ChIP-seq (ENCFF000BAO) (Davis et al., 2017[12]). ChIP-seq analysis was performed as previously described (Salmerón-Bárcenas et al., 2021[48]). We downloaded processed data corresponding to bigwig files of H3K27me3 (ENCFF958BAN) (Davis et al., 2017[12]), CTCF (ENCFF799KLZ) (Lee et al., 2020[29]; Lou et al., 2020[32]; Zhang et al., 2020[65]), H2AFZ (ENCFF532VF) (Zhang et al., 2020[65]), POLR2A (ENCFF959MZN) (Lee et al., 2020[29]; Lou et al., 2020[32]; Zhang et al., 2020[65]), H3K4me3 (ENCFF045NNJ) (Zhang et al., 2020[65]) and MYC (ENCFF609BPN) (Lou et al., 2020[32]; Zhang et al., 2020[65]) from HeLa-S3 cell line. ChIP-seq signal was visualized in deepTools2 (Ramírez et al., 2016[45]). The annotated genomic region of the EZH2 peaks was identified using the ChIPseeker package (Yu et al., 2015[63]). The bigwig files were visualized in the IGV genome browser (Robinson et al., 2011[47]).

### RNA-seq analysis

To determine whether the EZH2 enrichment is associated with gene expression levels in HeLa-S3, we analyzed RNA sequencing (RNA-seq) data available in ENCODE. Raw data (ENCFF000FOM/ ENCFF000FOV and ENCFF000FOK/ ENCFF000FOY) (Lee et al., 2020[29]; Moore et al., 2020[38]) were downloaded. RNA-seq analysis was performed in the Galaxy platform (Afgan et al., 2018[1]) as previously described (Salmerón-Bárcenas et al., 2021[48]).

### TCGA data analysis

The differentially expressed genes between CC patients with high and low EZH2 levels were analyzed using data from TCGA-CESC (Chang et al., 2013[7]). Differential expression between “high EZH2 vs low EZH2” was performed using the TCGAbiolinks and DESeq2 tools (Colaprico et al., 2016[10]; Love et al., 2014[33]). Differentially expressed genes were defined as genes with an adjusted *p-value *<0.05 and a fold change of 1.5.

To evaluate the expression of genes associated with silencers we used the Xena platform (Goldman et al., 2020[22]). Gene expression was analyzed in CC and normal tissue samples from TCGA and GTEx datasets.

### Definition of EZH2-rich domains

We identified EZH2-rich domains from the HeLa-S3 cell line using EZH2 ChIP-seq data as described by Cai and colleagues (2021[4]). This analysis uses an in-house customized script that mimicked the signal calculation of the ROSE package (Lunardon et al., 2014[34]). Briefly, we used previously analyzed EZH2 ChIP-seq signal and peaks. The EZH2 peaks were stitched using a window size of 4 kb. Then, the rank-ordered signal was used for defining EZH2-rich domains.

### Repressive compartments 

In order to evaluate whether EZH2-rich domains are associated with B compartments (inactive and closed chromatin), we used the 4D Nucleome Data Portal (Dekker et al., 2017[13]). We downloaded processed data corresponding to *in situ* Hi-C from the HeLa-S3 cell line (4DNESCMX7L58) (Dekker et al., 2017[13]). The compartment signal was visualized in the IGV genome browser.

### Functional enrichment analysis

To determine the pathways and biological processes regulated by EZH2 we used the Enrichr database (Chen et al., 2013[8]). We considered a *p-value *<0.05 as statistically significant.

The processes regulated by EZH2 in TCGA-CESC patients were evaluated using the Gene Set Enrichment Analysis (GSEA) (Subramanian et al., 2005[55]).

The senescence score in cancer patients was analyzed using the online tool Cancer SENESCyclopedia (Jochems et al., 2021[24]).

### Data analysis and visualization

The visualization and statistical analysis were performed in the ggplot2 package of R (Wickham, 2009[60]). A *p-value *<0.05 was considered statistically significant.

## Results

### EZH2 overexpression in cervical cancer

**Figure 1:**
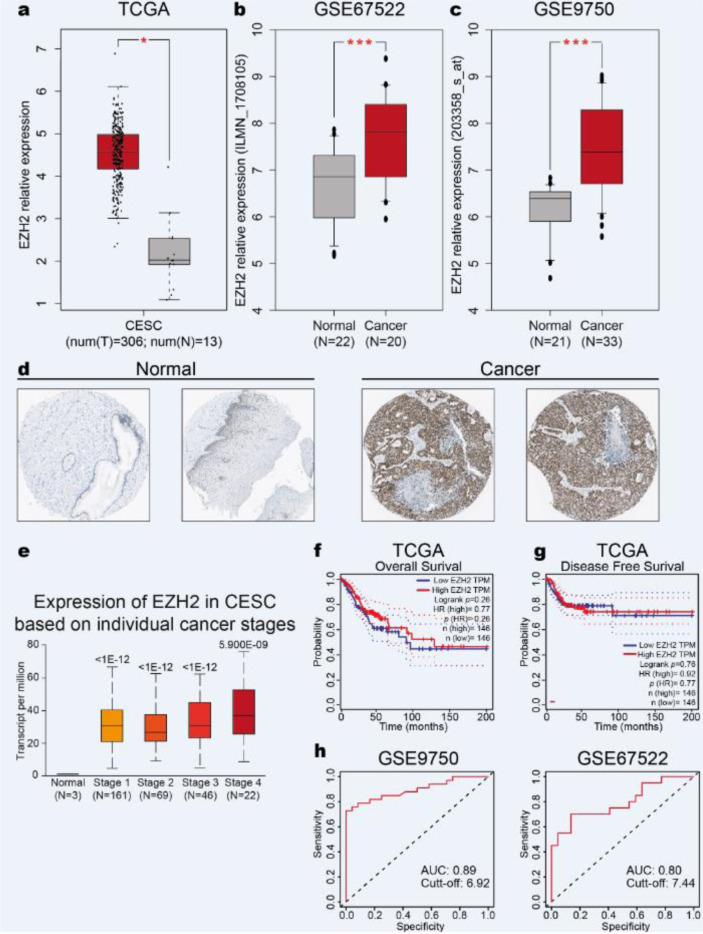
EZH2 expression is increased in cervical cancer. a) EZH2 expression in CC and normal samples from TCGA. One-way ANOVA, *p-value *˂0.05. b) EZH2 expression in CC and normal samples from the GSE67522 dataset. Student's *t*-test, *p-value *˂0.05. c) EZH2 expression in CC and normal samples from the GSE9750 dataset. Student's *t*-test,* p-value *˂0.05. d) EZH2 immunohistochemical level in CC and normal samples from The Human Protein Atlas database. Two representative images are depicted. e) EZH2 expression in CC stages and normal samples from TCGA. Student's *t*-test. *p-value* is shown. f) Kaplan-Meier OS curves of CC patients with high and low EZH2 expression. Log-Rank test, *p-value* = 0.26. g) Kaplan-Meier RFS curves of CC patients with high and low EZH2 expression. Log-Rank test, *p-value* = 0.76. h) ROC curves of EZH2 expression from GSE9750 and GSE67522 datasets. AUC>0.80

It has been reported that EZH2 overexpression has an oncogenic role in several types of cancer (Gan et al., 2018[20]; Kleer et al., 2003[25]; Varambally et al., 2002[58]). To determine whether EZH2 is deregulated in CC, we analyzed the EZH2 expression in CC samples compared with normal samples from TCGA and GTEx using the GEPIA database. We found an increase in EZH2 expression in CC (Figure 1a(Fig. 1)). To confirm this result, we analyzed the EZH2 expression in GSE67522 and GSE9750 datasets from the GEO database, and similarly, the results revealed an increase in EZH2 expression (Figure 1b, c(Fig. 1)). Then, we analyzed the EZH2 protein level in the HPA database. Consistent with the transcript data we found high levels of EZH2 protein in CC (Figure 1d(Fig. 1)).

We next sought to further evaluate whether the EZH2 overexpression is associated with CC progression. We found a significant correlation between EZH2 levels with the highest degree of malignancy in CC patients (Figure 1e(Fig. 1)). These results suggest that EZH2 overexpression is associated with CC progression.

To determine the prognostic value of EZH2 in CC, the overall survival (OS) and relapse-free survival (RFS) analysis according to EZH2 expression were performed through Kaplan Meier curves in CC tissue samples from the TCGA dataset using the GEPIA database. As shown in Figures 1f and 1g(Fig. 1), the OS and RFS in CC patients are not associated with high EZH2 expression. Then, we analyzed the diagnostic value of EZH2 through ROC analysis in CC tissue samples from GSE9750 and GSE67522 datasets using the easyROC tool. Interestingly, the results revealed an AUC: 0.89, sensitivity: 0.72, specificity: 1.00, *p*-value: 5.97E^-21^ and cut-off: 6.92 in GSE9750 dataset (Figure 1h(Fig. 1)). Similarly, an AUC: 0.80, sensitivity: 0.70, specificity: 0.86, *p*-value: 3.31E^-5^ and cut-off: 7.44 in GSE67522 dataset were observed (Figure 1h(Fig. 1)), suggesting that EZH2 could be a potential diagnostic value in CC.

### DNA methylation in the EZH2 promoter is decreased in cervical cancer

**Figure 2:**
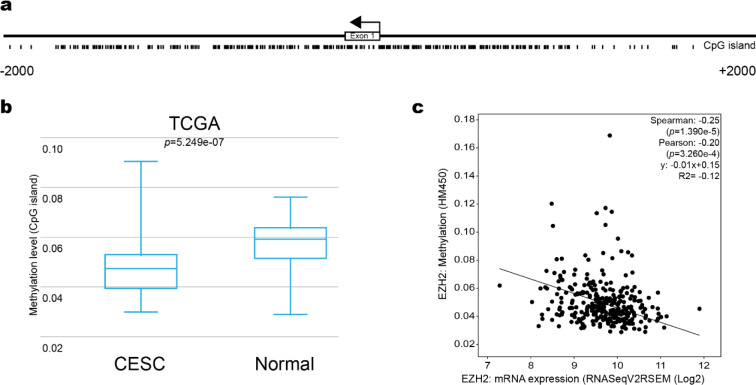
DNA hypomethylation in EZH2 promoter in cervical cancer. a) CpG islands at EZH2 promoters. The analyzed region corresponds to 2 kb around the TSS. b) Methylation level at EZH2 promoter in TCGA patients. Student's *t*-test, p*-value*=5.249e-07. c) Correlation between methylation levels and EZH2 expression in TCGA patients.

DNA hypomethylation has the potential to activate the gene expression in cancer cells (Das and Singal et al., 2004[11]). To determine whether DNA hypomethylation is associated with EZH2 overexpression in CC, we search a CpG island at the EZH2 promoter using the MethPrimer database. Figure 2a(Fig. 2) shows a CpG island present in the EZH2 promoter. Interestingly, we found DNA hypomethylation in the CpG island located in the EZH2 promoter in CC samples from TCGA (Figure 2b(Fig. 2)). Finally, to explore whether DNA hypomethylation promotes the EZH2 overexpression, we performed a correlation analysis between DNA methylation and EZH2 expression using the cBioportal database. This analysis showed a negative correlation between DNA methylation and EZH2 expression in CC samples (Spearman: -0.25 (*p*=1.390e-5), Pearson: -0.20 (*p*=3.260e-4) and R^2^= -0.12 (y: -0.01x+0.15)) (Figure 2c(Fig. 2)). Altogether, these results suggest that the DNA hypomethylation at the EZH2 promoter could be involved in its overexpression in CC.

### EZH2 enrichment at promoters in HeLa-S3

**Figure 3:**
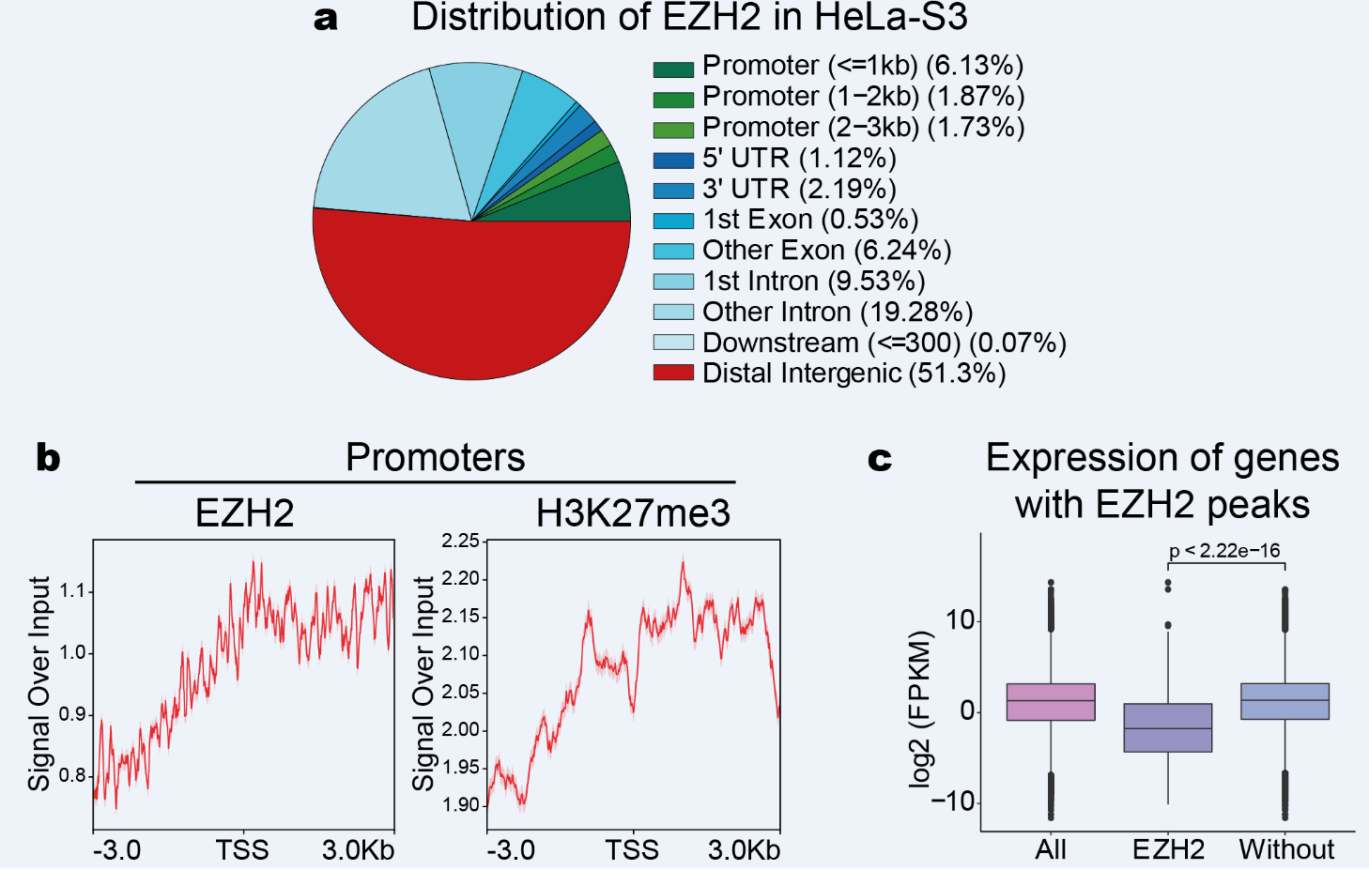
EZH2 enrichment at promoters in HeLa-S3 promotes transcriptional repression. a) Genomic annotation of EZH2 peaks in HeLa-S3. b) Profile plots of EZH2 and H3K27me3 enrichment around the TSS. The signal intensity of EZH2 and H3K27me3 are shown concerning the input. c) Box plot of the transcription levels of genes with EZH2 enrichment at promoters in HeLa-S3. Wilcoxon test, *p-value *<2.22e-16.

It has been reported that PRC2 is bound to promoters of repressed genes by catalyzing the histone mark H3K27me3 which is a hallmark of facultative heterochromatin (Meng et al., 2020[37]). To determine the EZH2 enrichment in HeLa-S3, we analyzed ChIP-seq data. We identified 40,652 EZH2 peaks (Supplementary data, Table 1), the annotation peaks show EZH2 enrichment at promoters (~10.0 %) and distal regions (51.3 %) in HeLa-S3 (Figure 3a(Fig. 3)). As expected, EZH2 peaks are associated with high H3K27me3 enrichment at promoter regions (Figure 3b(Fig. 3)). Our analysis suggested that EZH2 could have a key role in transcriptional repression. To address this question broadly, we evaluate the transcription level associated with EZH2 at promoter regions. We detected a low expression of genes associated with EZH2 in HeLa-S3 cells (Wilcoxon test; *p*-value <2.22e-16) (Figure 3c(Fig. 3)). This result is consistent with the previous reports that identified EZH2 as a transcriptional repressor through induction of repressive histone methylation on H3K27me3 (Cao and Zhang et al., 2004[5]; Varambally et al., 2002[58]; Yang et al., 2009[62]).

### EZH2-rich domains promote transcriptional repression in cervical cancer

**Figure 4:**
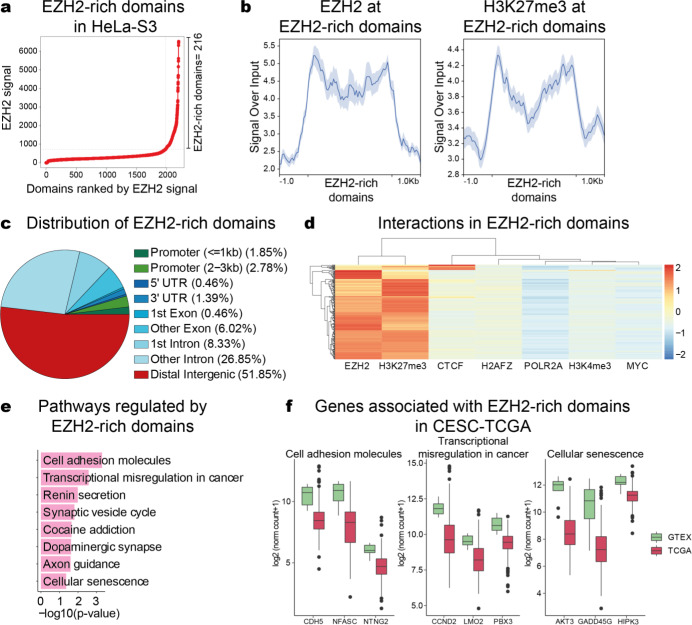
EZH2-rich domains promote transcriptional repression in cervical cancer. a) EZH2-rich domains in HeLa-S3 cells. b) Profile plots of EZH2 and H3K27me3 enrichment at EZH2-rich domains in HeLa-S3 cells. The signal intensity of EZH2 and H3K27me3 are shown concerning the input. c) Genomic annotation of EZH2-rich domains in HeLa-S3. d) Hierarchical clustering of EZH2-rich domains enriched with EZH2, H3K27me3, CTCF, H2A.Z, POLR2A, H3K4me3, and MYC in HeLa-S3. e) Functional annotation of genes near to EZH2-rich domains. The most significant pathways are shown. We considered a *p-value *<0.05 as statistically significant. f) Expression of genes associated with EZH2-rich domains in TCGA and GTEx patients. We analyzed genes near EZH2-rich domains in HeLa-S3 associated with cell adhesion molecules, transcriptional misregulation in cancer, and cellular senescence.

Silencers are DNA regions that promote gene repression (Cai et al., 2021[4]). Yichao Cai and colleagues (2021[4]) identified that H3K27me3-rich regions contain silencers that can repress target genes (Cai et al., 2021[4]). We hypothesize that EZH2-rich domains are also associated with these silencer regions. To test the above hypothesis, we defined the EZH2-rich domains by following Cai's method (Cai et al., 2021[4]). The classification of clustered peaks by average EZH2 signal levels allowed the definition of 216 EZH2-rich domains in HeLa-S3 cells (Figure 4a(Fig. 4) and Supplementary data, Table 2). These EZH2-rich domains are characterized by a high EZH2 and H3K27me3 enrichment (Figure 4b(Fig. 4)). Interestingly, the EZH2-rich domains are located mainly in distal regions (51.85 %) (Figure 4c(Fig. 4)), suggesting that EZH2-rich domains could regulate gene expression through long-range interactions.

Furthermore, we analyzed the epigenetic marks and transcription factors binding to the EZH2-rich domains. CTCF enrichment is present in these regions (Figure 4d(Fig. 4)) suggesting an association between EZH2-rich domains and chromatin interactions. H2A.Z enrichment is also associated with EZH2 as previously reported (Figure 4d(Fig. 4)) (Ku et al., 2012[27]). As expected, the EZH2-rich domains have no association with active marks such as RNApol II, H3K4me3, and MYC (Figure 4d(Fig. 4)).

To evaluate the biological role of EZH2-rich domains in cervical carcinogenesis, we analyzed the associated genes. KEGG pathway enrichment analysis revealed enrichment of essential pathways for oncogenic processes such as cell adhesion molecules, transcriptional misregulation in cancer, and cellular senescence (Figure 4e(Fig. 4)). Interestingly, we found transcriptional repression of genes associated with these pathways in TCGA and GTEx patients (Figure 4f(Fig. 4)). These results suggest the presence of EZH2-rich domains in the HeLa-S3 genome, which is associated with repression marks, in addition, suggest that the activity of EZH2-rich domains could allow transcriptional repression in CC.

**Figure 5:**
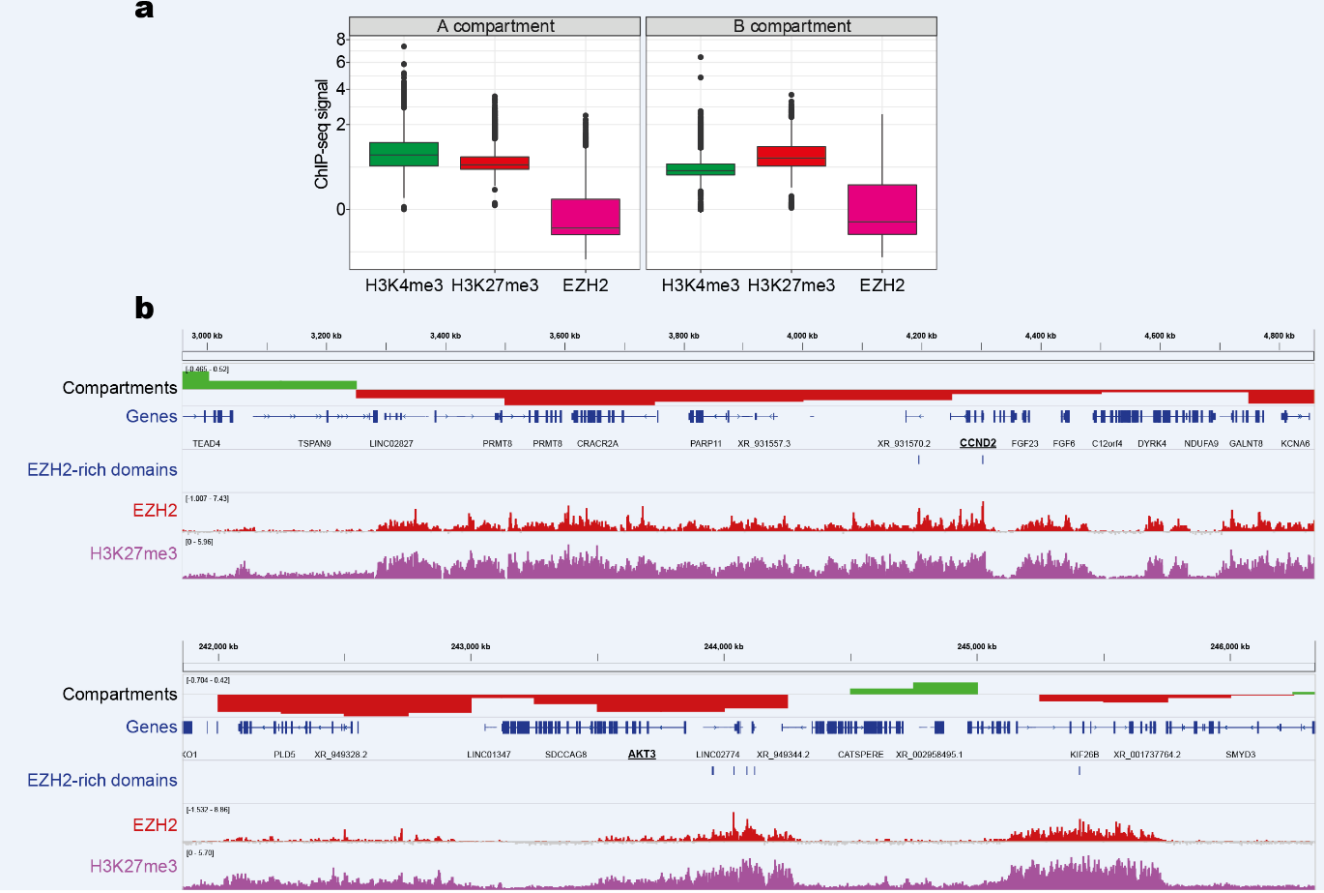
EZH2-rich domains are associated with repressive compartments in HeLa-S3. a) EZH2 enrichment at A/B compartments in HeLa-S3 cells. A/B compartments were identified with *in situ* Hi-C from the HeLa-S3 cell line using the 4D Nucleome Data Portal. b) IGV tracks of the A/B compartments and EZH2-rich domains in the CCND2 and AKT3 locus. The tracks of EZH2 and H3K27me3 are shown. The region analyzed shows clear compartmentalization between B compartments and EZH2-rich domains.

H3K27me3 domains have been reported to be associated with repressed compartments (Yuan et al., 2021[64]). To investigate whether EZH2-rich domains are located in inactive and closed chromatin, we analyzed the A/B compartments determined by Hi-C using the 4D Nucleome Data Portal. As expected, the EZH2-rich domains have high enrichment in B compartments (Figure 5a(Fig. 5)). In particular, we found EZH2-rich domains associated with CCND2 and AKT3 within the B compartments in HeLa-S3 (Figure 5b(Fig. 5)). This result raises the possibility that EZH2-rich domains within the B compartments could promote gene repression via chromatin interactions. 

### High EZH2 expression is associated with repression of senescence in cervical cancer

**Figure 6:**
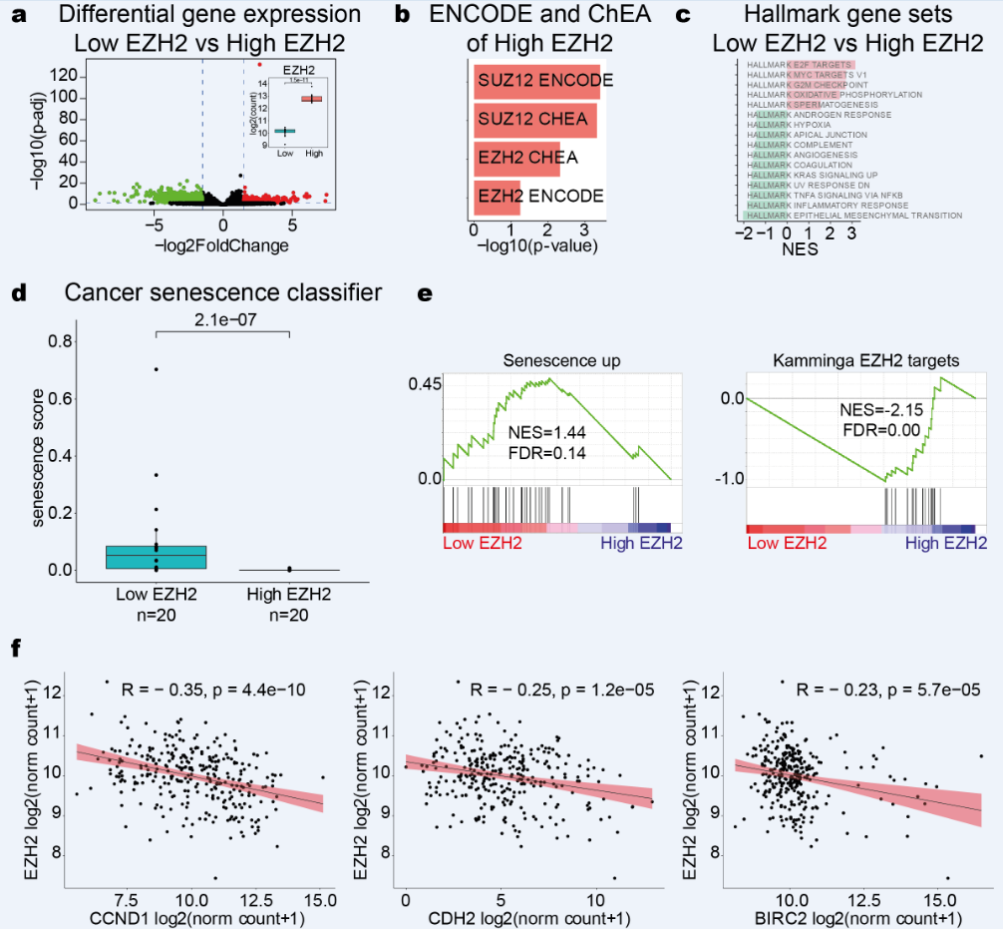
High EZH2 expression reduces senescence in cervical cancer. a) Volcano plot shows the differentially expressed genes between CC patients with low EZH2 and high EZH2. An adjusted *p-value *<0.05 and an FC of 1.5 were considered statistically significant. b) TFs associated with genes regulated by high EZH2. A p-value <0.05 was considered statistically significant. c) The top hallmark GSEA processes enriched in low EZH2 (green) and high EZH2 (red). NOM *p-value *<0.05 were considered statistically significant. d) Senescence score between CC patients with low EZH2 and high EZH2. Wilcoxon test, *p-value *<2.1e-07. e) GSEA plots of the differentially expressed genes between CC patients with low EZH2 and high EZH2. The processes correspond to genes enriched in cellular senescence. f) Correlation plots between EZH2 with CCND1, CDH2, and BIRC2 in TCGA. A *p-value *<0.05 was considered statistically significant.

EZH2 is associated with the carcinogenic process in several types of cancer (Duan et al., 2020[15]). To investigate the biological pathways associated with the EZH2 increase in CC patients, we evaluated the differentially expressed genes between CC patients with high EZH2 expression (20 patients) and low EZH2 expression (20 patients) using TCGA data. We found 423 genes associated with high EZH2 expression and 1,420 genes associated with low EZH2 expression in CC (adjusted *p-value*<0.05; FC of 1.5; Figure 6a(Fig. 6) and Supplementary data, Table 3). In addition, ENCODE and ChEA Consensus analysis identified that the genes associated with high EZH2 expression are regulated by SUZ12 and EZH2 (Figure 6b(Fig. 6)). GSEA analysis showed enrichment of processes associated with E2F targets, MYC targets, G2M checkpoint, and oxidative phosphorylation in high EZH2 expression (NOM *p-value *<0.05) (Figure 6c(Fig. 6)). On the other hand, processes associated with epithelial-mesenchymal transition, inflammatory response, and TNF-α signaling are associated with low EZH2 expression (NOM *p-value *<0.05) (Figure 6c(Fig. 6)).

It has been reported that EZH2 downregulation promotes senescence by allowing DNA damage prior to a reduction in H3K27me3 levels (Ito et al., 2018[23]). Interestingly, high EZH2 expression was associated with a low senescence score compared with low EZH2 expression (Wilcoxon test, *p-value *<2.1e-07) (Figure 6d(Fig. 6)). GSEA analysis reinforced this result (Figure 6e(Fig. 6)), suggesting that EZH2 overexpression in CC could be associated with overcoming the senescent phenotype allowing the expression of genes associated with proliferation (E2F targets, MYC targets, G2M checkpoint). Besides, the genes associated with senescence CCND1, CDH2, and BIRC2 have a negative correlation with EZH2 in TCGA-CESC patients (Figure 6f(Fig. 6)).

**Figure 7:**
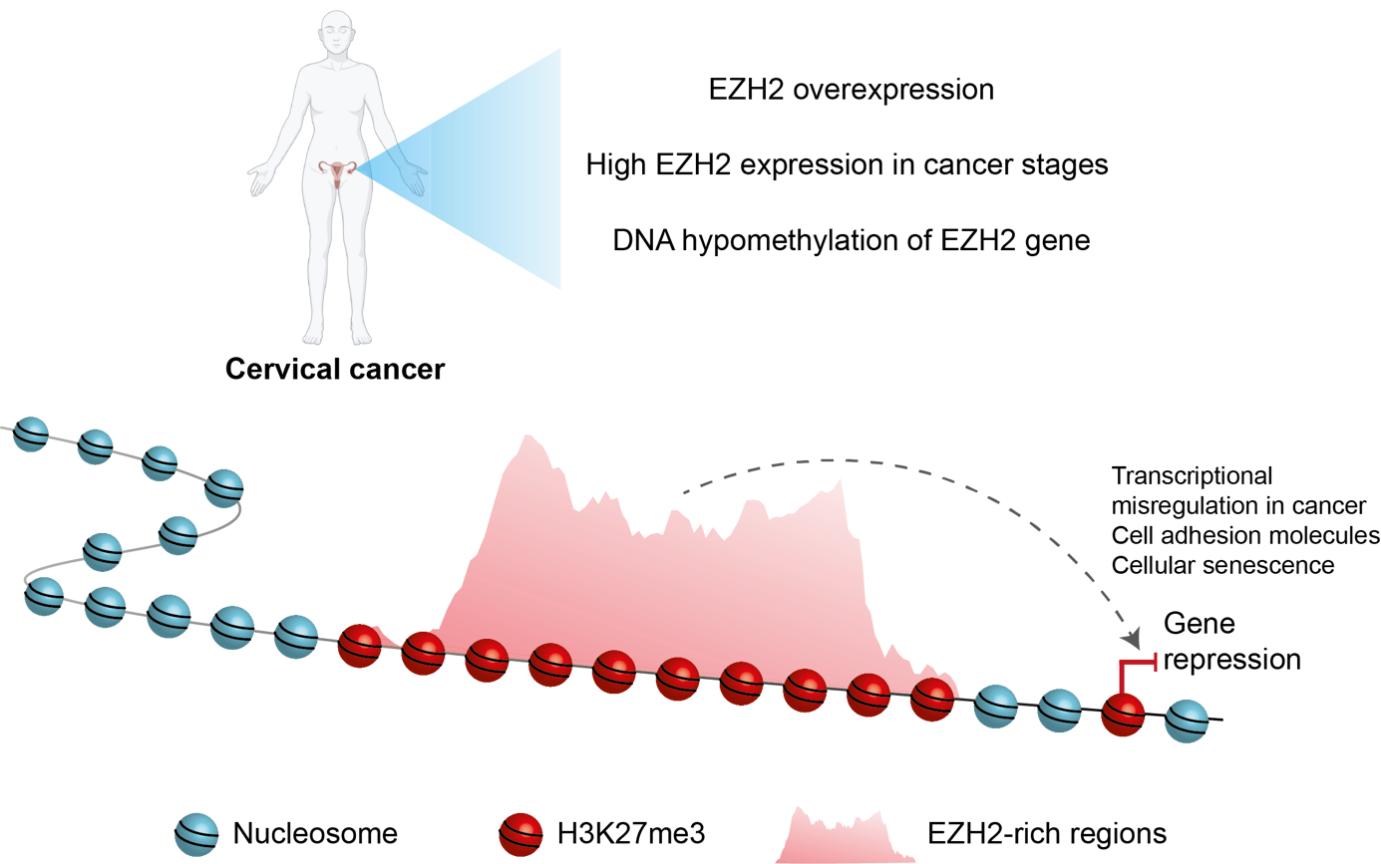
Proposed model for EZH2-rich domains in cervical cancer. EZH2 is overexpressed in CC patients. EZH2 overexpression is associated with DNA hypomethylation of its promoter. EZH2 is enriched at promoters where it regulates transcriptional repression. Besides, EZH2 generates domains enriched with H3K27me3. These domains are associated with repressive compartments, which allow the silencing of target genes. Finally, EZH2 is associated with the repression of cancer-associated genes in CC, mainly genes associated with cellular senescence.

In summary, these findings support a model in which EZH2-rich domains promote gene repression allowing oncogenic processes in CC (Figure 7(Fig. 7)). 

## Discussion

In this study, we analyzed the role of EZH2 to reveal the mechanism associated with gene repression through chromatin structure in CC. EZH2 is associated with chromatin domains enriched with H3K27me3, which facilitated transcriptional silencing in HeLa-S3 cells and CC patients. These EZH2-rich domains could start the deposition of H3K27me3 promoting a silencer function through interactions within repressive compartments in CC.

Here, we found an EZH2 overexpression in CC samples. These results are consistent with those reported by Yueyang Liu and colleagues (2014[31]) who demonstrate high EZH2 levels by immunohistochemistry in 68.3 % of CC samples. Furthermore, the results show a positive correlation with the proliferation marker Ki-67 in 57.4 % of the samples (Liu et al., 2014[31]). These results allow us to suggest that EZH2 overexpression could be associated with carcinogenesis and proliferation and could represent a predictor of prognosis in CC. Besides, we identified DNA hypomethylation at EZH2 promoter in CC patients. Interestingly, Schäfer and colleagues (2016[49]), found DNA hypermethylation at EZH2 promoter in childhood acute lymphoblastic leukemia (Schäfer et al., 2016[49]), suggesting that DNA methylation is important to EZH2 dysregulation in several types of cancer. Therefore, our results suggest an oncogenic role of EZH2 in CC.

EZH2 trimethylates H3K27 promoting the PRC1 binds to H3K27me3 and generates the histone mark H2AK119Ub1, allowing the chromatin compaction and gene repression (Cao and Zhang et al., 2004[5]). We identified EZH2 enrichment at promoters in HeLa-S3 cells, which correlates with H3K27me3 and transcriptional repression. It has been reported that EZH2 and H3K27me3 are implicated in proliferation and carcinogenesis (Gannon et al., 2013[21]; Margueron et al., 2008[36]). For instance, in prostate cancer, EZH2 and H3K27me3 are present at the promoter of tumor suppressor gene ID4, avoiding its expression (Chinaranagari et al., 2014[9]). Therefore, these data suggest that EZH2 recruitment promotes transcriptional repression through H3K27me3 at promoters in HeLa-S3.

We found high EZH2 enrichment at distal intergenic regions in HeLa-S3 cells. Based on these observations, we hypothesized that EZH2 could be regulating gene silencing through chromatin interactions. Silencers have been reported to be regulatory elements of gene repression through interaction with target genes (Cai et al., 2021[4]; Ngan et al., 2020[40]). Previously, it has been identified that H3K27me3-rich regions are associated with silencers and can regulate gene expression via looping (Cai et al., 2021[4]). Based on these observations, we found that EZH2 produces chromatin domains (EZH2-rich domains) associated with H3K27me3 enrichment and gene repression. EZH2-rich domains have an occupancy of the architectural protein CTCF, which suggests that EZH2-rich domains could be regulating transcriptional repression through chromatin interactions in HeLa-S3. In line with these results, previous reports have shown that H3K27me3-rich regions can repress transcription via chromatin interactions by the association of several architectural proteins (CTCF, RAD21, and SMC3) (Cai et al., 2021[4]). Similarly, the histone variant H2A.Z is also present in EZH2-rich domains. H2A.Z mono-ubiquitylation has co-occupancy with H3K27me3 and depleted H3K4me3 at non-expressed genes (Ng et al., 2019[39]). Strikingly, H2A.Z mono-ubiquitylation copurify with proteins involved in the 3D organization of the genome, such as CTCF and cohesin (Ng et al., 2019[39]). Collectively, these observations suggest that EZH2-rich domains may have a regulatory network with architectural proteins and histone variants to promote the repression of chromatin interactions in HeLa-S3, thus its exploration in cervical carcinogenesis is required.

Pathway analyses revealed that EZH2-rich domains regulate genes associated with processes involved in cancer such as cell adhesion molecules, transcriptional misregulation in cancer, and cellular senescence in HeLa-S3. Inspection of gene expression of these genes in CC patients revealed a significant reduction compared to healthy patients. Based on these observations, our analysis suggests that EZH2-rich domains could be functional in cell lines and CC patients. Therefore, elucidating the silencing role of EZH2-rich domains in CC patients would be an important future direction for research.

*In situ* Hi-C analysis revealed a high density of interactions in H3K27me3 (Cai et al., 2021[4]). Consistent with this report, EZH2-rich domains are slightly enriched in B compartments. This result implies that EZH2-rich domains could be involved in transcriptional repression within repressive compartments in the HeLa-S3 genome. Additional studies are needed to validate the crosstalk between EZH2-rich domains and chromatin interactions and how this affects gene expression levels. However, one piece of data supporting this notion comes from the EZH2 inhibition which disturbs the chromatin interactions between H3K27me3-rich regions and target genes (Cai et al., 2021[4]). Recent studies have shown that silencer depletions allow tumor growth inhibition (Cai et al., 2021[4]). Here we show evidence to suggest that EZH2-rich domains regulate genes associated with processes such as cellular senescence in HeLa-S3. Consistent with these results, CC patients with high EZH2 levels are characterized by an increase in genes associated with cell proliferation (E2F targets, MYC targets, G2M checkpoint). Strikingly, high EZH2 levels promote a reduction in the senescent phenotype. Based on our data, we speculate that EZH2 overexpression promotes a proliferative profile necessary to avoid senescence, allowing the carcinogenic process. These data are consistent with previous studies which demonstrated that downregulation of EZH2 allows senescence through two mechanisms, 1) EZH2 and H3K27me3 depletion initiate a DNA damage response, and 2) H3K27me3 loss allows the activation of p16 expression and senescence-associated secretory phenotype. Interestingly, in our system, we found an inverse correlation between EZH2 levels and genes associated with cellular senescence such as CCND1 and CDH2, two genes reduced in the senescent phenotype (Laphanuwat et al., 2018[28]; Waldera Lupa et al., 2015[59]). More studies are needed to know how EZH2-rich domains directly regulate the expression of genes associated with senescence in CC progression.

The role of silencers in cancer has been poorly studied. Our study contributes to the understanding of its characteristics and mechanisms associated with transcriptional regulation in CC biology. Furthermore, EZH2 represents a potential target for CC therapy. In line with this, Ding and colleagues (2015[14]), demonstrated that the EZH2 inhibitor, GSK343, significantly suppresses the proliferative phenotype and the mesenchymal-epithelial transition in CC cells (Ding et al., 2015[14]). Besides, currently, there are different types of EZH2 inhibitors and some of them are in clinical trials for different types of cancer (Duan et al., 2020[15]). Thus, the EZH2 inhibition in CC could represent a potential therapeutic target to reverse the invasive phenotype.

## Conclusions

Our work suggests an oncogenic role of EZH2 in CC by regulating gene silencing at promoter and EZH2-rich domains level. In addition, our analysis suggests that EZH2-rich domains promote the transcriptional repression of genes associated with cancer and cellular senescence, promoting disease progression.

## Declaration

### Competing interests

The authors declare that they have no competing interests.

### Acknowledgments

This work was supported by the Consejo Nacional de Ciencia y Tecnología fellowship 396917 to Pedro A. Ávila-López. 

### Authors' contributions

EGSB and PAAL: Data curation and bioinformatic analysis. EGSB and PAAL: Writing original draft. AEZG, JOO, and FITR: Writing, reviewing, and editing. All authors read and approved the final manuscript.

## Supplementary Material

Supplementary data
